# Unraveling Transformative Effects after tDCS and BCI Intervention in Chronic Post-Stroke Patient Rehabilitation—An Alternative Treatment Design Study

**DOI:** 10.3390/s23239302

**Published:** 2023-11-21

**Authors:** Jéssica P. S. Lima, Leticia A. Silva, Denis Delisle-Rodriguez, Vivianne F. Cardoso, Ester M. Nakamura-Palacios, Teodiano F. Bastos-Filho

**Affiliations:** 1 Postgraduate Program in Biotechnology, Federal University of Espirito Santo (UFES), Vitoria 29047-105, Brazil; jpaola.fisio@gmail.com (J.P.S.L.); viviannefc@gmail.com (V.F.C.); teodiano.bastos@ufes.br (T.F.B.-F.); 2 Postgraduate Program in Electrical Engineering, Federal University of Espirito Santo (UFES), Vitoria 29075-910, Brazil; araujos.leticia@gmail.com; 3 Postgraduate Program in Neuroengineering, Edmond and Lily Safra International Institute of Neurosciences, Macaiba 59288-899, Brazil; 4 Laboratory of Cognitive Sciences and Neuropsychopharmacology, Federal University of Espírito Santo, Vitoria 29040-090, Brazil; emnpalacios@gmail.com

**Keywords:** neuromuscular disability, robotic devices, cortical excitability, mental practice training

## Abstract

Stroke is a debilitating clinical condition resulting from a brain infarction or hemorrhage that poses significant challenges for motor function restoration. Previous studies have shown the potential of applying transcranial direct current stimulation (tDCS) to improve neuroplasticity in patients with neurological diseases or disorders. By modulating the cortical excitability, tDCS can enhance the effects of conventional therapies. While upper-limb recovery has been extensively studied, research on lower limbs is still limited, despite their important role in locomotion, independence, and good quality of life. As the life and social costs due to neuromuscular disability are significant, the relatively low cost, safety, and portability of tDCS devices, combined with low-cost robotic systems, can optimize therapy and reduce rehabilitation costs, increasing access to cutting-edge technologies for neuromuscular rehabilitation. This study explores a novel approach by utilizing the following processes in sequence: tDCS, a motor imagery (MI)-based brain-computer interface (BCI) with virtual reality (VR), and a motorized pedal end-effector. These are applied to enhance the brain plasticity and accelerate the motor recovery of post-stroke patients. The results are particularly relevant for post-stroke patients with severe lower-limb impairments, as the system proposed here provides motor training in a real-time closed-loop design, promoting cortical excitability around the foot area (Cz) while the patient directly commands with his/her brain signals the motorized pedal. This strategy has the potential to significantly improve rehabilitation outcomes. The study design follows an alternating treatment design (ATD), which involves a double-blind approach to measure improvements in both physical function and brain activity in post-stroke patients. The results indicate positive trends in the motor function, coordination, and speed of the affected limb, as well as sensory improvements. The analysis of event-related desynchronization (ERD) from EEG signals reveals significant modulations in Mu, low beta, and high beta rhythms. Although this study does not provide conclusive evidence for the superiority of adjuvant mental practice training over conventional therapy alone, it highlights the need for larger-scale investigations.

## 1. Introduction

Stroke is a clinical condition that consists of a sudden loss of focal neurological function due to an infarction or hemorrhage in the central nervous system (CNS) [1]. The consequences of a stroke can vary depending on the injury severity and its location in the brain, including sudden unilateral weakness, numbness, ataxia, vertigo, loss of balance, and difficulty walking [2]. Motor function restoration after a stroke is a complex process that involves neuroplasticity, spontaneous recovery, and induced recovery as the effects of therapeutic interventions [3].

The social and economic costs directly related to neuromuscular disabilities, such as stroke, are significant. This includes health and rehabilitation services, specific projects aimed at the labor market, education and professional training, social benefits, the distribution of assistive devices, and urban mobility [4]. The relatively low cost of tDCS devices along with the potential beneficial effects, combined with its safety, simplicity, painlessness, and easy portability, make it an ideal candidate to be used as an additional clinical tool before or during therapy to intensify neuroplastic changes and recover motor function. Various neuromodulation methods have been proposed to induce cortical plasticity and promote motor recovery after stroke [5]. For instance, the use of low-cost robotic devices for neuromuscular rehabilitation may optimize therapy and reduce the cost of the rehabilitation process [6].

Studies conducted by Mohammadi, A., 2016 [7] have shown that tDCS for motor training improves neuroplasticity in patients with neurological diseases or disorders. This modern non-invasive brain stimulation technique is used to generate cortical temporary excitability [8,9]. The main advantage of applying tDCS may come from its use associated with conventional therapies, as this is a path to improved and enlarged motor gains. tDCS activates a change in the dysfunctional excitability pattern so that physical therapy can model the functional pattern of the cortex activity with the activation of specific neural networks [10]. However, while research has demonstrated that tDCS has the potential to improve upper-limb motor function in post-stroke patients when combined with intensive physical exercises [5,11], few studies have been reported for the lower limbs.

Another type of innovative tool used to help generate neuroplasticity is a brain-computer interface (BCI). BCI is a communication system that measures the CNS activity and converts it into artificial commands to control end-applications [12]. Typically, a BCI enables user-system interaction through the classification of observable brain activity in electroencephalographic (EEG) signals. The primary mode of interaction involves the detection of deliberate changes in sensorimotor rhythms during motor imagery (MI), which can play a crucial role for post-stroke patients with severe neural impairments [13], as they are still able to imagine movements of the paretic limb, even in the absence of real movements [14].

MI is defined as a dynamic event during which an individual mentally reproduces an action, and it encompasses two fundamental types: kinesthetic motor imagery (KMI) and visual motor imagery (VMI). KMI involves simulating movement sensations without actual execution, and VMI is primarily centered around visualizing movement execution. Both KMI and VMI engage overlapping neural networks found in regions like the primary motor cortex, supplementary motor areas, somatosensory cortex, and cerebellum [15]. Our BCI measures brain waves linked to a pedaling KMI task, converting it into artificial commands to control a motorized pedal (MP), which is synchronized through an inertial measurement unit (IMU) with a VR environment [16]. The VR may also help an individual to imagine lower-limb movements, which is fundamental for MI [17].

Notably, previous studies utilizing tDCS in association with MI-BCI have primarily focused on lower-limb rehabilitation using costly equipment or have centered on studies with healthy individuals. For instance, the investigation by Chew [11] on the impact of MI-BCI coupled with robotic arm training and tDCS priming, alongside Rodriguez-Ugarte’s [18] study, enhanced real-time cortical excitability but primarily focused on the upper-limb rehabilitation of healthy subjects. This underscores the unique focus and innovation of the present study, addressing a gap in the scientific research by exploring the efficacy of integrating tDCS, MI-BCI, and VR for lower-limb post-stroke patient recovery. Consequently, this research extends novel insights with the potential advantages of combining these technologies in lower-limb post-stroke rehabilitation, offering a fresh and invaluable perspective within the field of neurological rehabilitation.

The proposed method integrates cutting-edge technologies including tDCS, MI-BCI, VR, and a customized MP to facilitate the lower-limb rehabilitation of post-stroke patients. This innovative system also introduces a novel approach to BCI calibration, combining EEG signals from both MI and actual movements [19], fostering a real-time closed-loop system for neural rehabilitation. Furthermore, the study highlights the potential benefits and cost-effectiveness of tDCS in inducing cortical plasticity for motor recovery in lower-limb rehabilitation, aiming to mitigate neuromuscular disabilities and associated societal costs. This research serves to bridge the gap in exploring lower-limb post-stroke recovery, offering insights into the potential therapeutic advantages of combining tDCS with conventional therapies and low-cost neuromodulation techniques.

## 2. Materials and Methods

### 2.1. Patient

The patient in our research was a 73-year-old male, suffering from right hemiparesis of a subacute (2 months) hemorrhagic post-stroke, who was followed by a multidisciplinary rehabilitation team composed of clinical staff and engineers for 3 weeks.

The inclusion criteria to be included in this research were as follows: one participant in the subacute phase to chronic post-stroke; stable medical condition; good understanding and ability to follow instructions according to the score on the Mini-Mental State Examination (MMSE); and good tolerance when standing using or not using support. The exclusion criteria were scores above 2 on the Ashworth Scale; severe aphasia; severe perceptual problems or other neurological conditions; severe osteoporosis; skin lesions at the electrode site; very limited visual ability; more than one stroke episode; and no signed informed consent for experiments.

### 2.2. Study Design

This study adopted an ATD (quasi-experimental), as suggested by Slijper in 2014 [20], to ensure the safety regarding cross-infection risk among equipment users. Our study was a double-blind investigation conducted in a controlled environment, ensuring safety and privacy for the participant. The ATD used here was a single-subject design, beginning with a baseline phase to characterize the initial dependent variables and implement interventions while continuously collecting data. The ATD allows the comparative analysis of treatment conditions and their effects. Stringent measures for experimental rigor include an established implementation schedule and careful consideration of confounding factors. This design, categorized as a Level I study, encompasses various approaches, such as randomized controlled N-of-1 studies and multiple-baseline designs. Achieving Level I status involves replication across multiple participants or settings while maintaining randomization, facilitating broader applicability and causal inferences.

This research was approved by the Research and Ethical Committees of the UFES/Brazil (registry number CAAE: 46099421.9.0000.5542) and conducted in accordance with the Declaration of Helsinki.

### 2.3. Protocol

**Figure 1:**
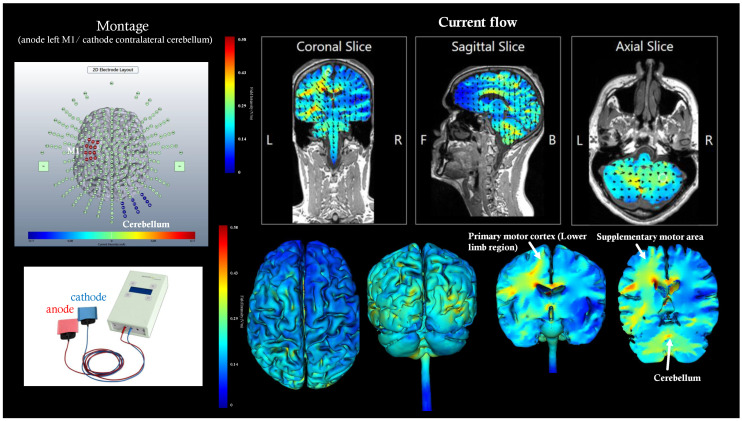
Assembly modeling with the tDCS equipment used in our study, with the anode positioned over M1 and the cathode positioned over the cerebellum of the other hemisphere.

In our protocol, before the use of the BCI, tDCS was applied for 20 min, with an intensity of 2 mA, following the dual-tDCS model, with the anode positioned over M1 of the affected hemisphere and the cathode positioned over the cerebellum of the contralateral hemisphere (Figure 1). Regarding the sham procedure, this was applied with the same equipment and electrode placement; however, the subject was only subjected to an initial and momentary dose.

### 2.4. Brain-Computer Interface

**Figure 2:**
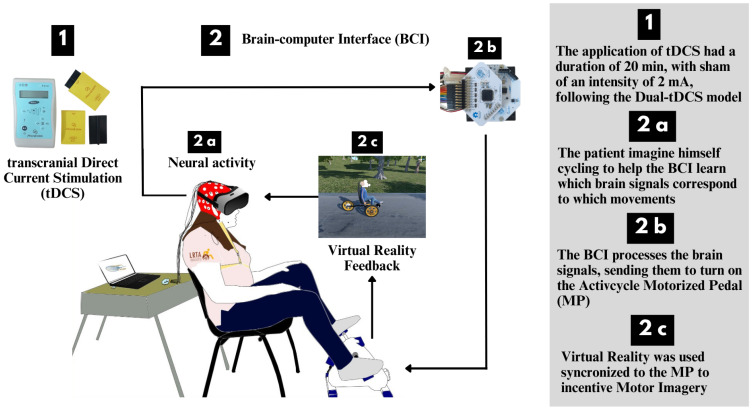
Methods: (1) The subject is subjected to brain stimulation through transcranial direct current stimulation (tDCS). (2a) The brain-computer interface (BCI) uses electroencephalography signals to control the on/off and velocity of the motorized pedal. (2b) The BCI processes the brain signals, sending them to turn on the motorized pedal. (2c) The virtual reality (VR) device is used to active motor imagery (MI).

The system developed here is a novel MI-BCI [19] integrated with VR and a customized MP (7101 Activcycle Motorized Pedal Exerciser, Exerpeutic, USA) using an end-effector pedal. The system comprises four main sub-systems, namely EEG signal acquisition, signal processing and MI classification, an MP with a communication and control interface board, and a VR device (Figure 2).

The EEG signal acquisition is achieved using the OpenViBE platform, with an EEG cap used to acquire brain signals through Ag/AgCl electrodes at 8 locations (FC1, FC2, C3, C4, Cz, CP1, CP2, and Pz) positioned according to the International 10–20 system. The A1 Ground (GND) and A2 reference (REF) electrodes are placed on the left and right ears, respectively. The acquired EEG signals are then processed and classified to recognize MI patterns related to pedaling movements. The MI classification is an essential step in translating the user’s intentions into control commands for the MP.

The MP is designed to be controlled by the user’s brain signals, where a communication and control interface board facilitates the transmission of classification outputs obtained from the MI recognition phase. In this way, users can effectively command the MP using their brain activity.

To provide an immersive experience, the system incorporates a VR device. The movement of the MP is captured through an IMU with a Bluetooth connection, allowing real-time data transfer to a specific computer running the VR environment, created by us. This VR device offers visual feedback to the user, creating an interactive and engaging virtual environment during the rehabilitation process, in order to ensure the user’s motivation. In the virtual environment, there is a first-person avatar seated on a tricycle and a real customized MP.

The system employs a script developed in Python3 (Version 3.8.0, Python Software Foundation, Wilmington, DE, USA) and an MQTT protocol for efficient communication between the various components. Notably, OpenViBE (Version 3.5.0, Free Software Foundation, Boston, MA, USA), Python3, and the MQTT broker operate simultaneously on a single laptop, streamlining the system’s performance.

The MI-BCI proposed in this study is divided into two phases: the calibration phase and the online phase. During the calibration phase, EEG signals collected during rest, motor imagery, and passive pedaling are processed to create a robust classification model for MI recognition. Subsequently, in the online phase, this model is employed, enabling the user to command the customized motorized pedal accurately.

In summary, the developed system composed of MI-BCI and VR with a pedal end-effector offers a promising approach to neurological rehabilitation. By leveraging brain signals to control the MP and providing real-time visual feedback through VR, the system aims to enhance motor function and facilitate effective rehabilitation processes. The integration of these components on a single laptop ensures efficiency and practicality in its implementation.

The motor training was conducted immediately after the tDCS application. The pedaling exercise lasted 20 min, and the training sessions were carried out at the same time of the day, five times a week, for 3 weeks.

### 2.5. Functional and Somatosensory Outcomes

In our protocol, the subject was subjected weekly, and at the end of the training period, to functional and somatosensory assessments by the same evaluator. For function evaluation, we used the following metrics: Fugl-Meyer [21], Mini Balance Evaluation Systems Test (MiniBEST Test) [22], and 10-m walk test [23].

We also used the Semmes Weinstein Monofilaments to evaluate the subject’s sensitivity to crude to fine touch in the dermatomes. This type of test is considered an easy-to-use and inexpensive touch threshold test, and it is generally used by clinicians to evaluate sensory disorders in neuropathic diseases. This test is carried out using flexible nylon monofilaments of equal length with different diameters; the thicker the filament becomes, the more pressure is necessary to bend the filament. These filaments are labeled to give a linear scale of perceived intensity, with weights ranging between 0.2 and 300 g [24]. As there is no standard regarding specific localization to conduct this test, we used locations representing peripheral nerves.

All functional parameters of this study produced quantitative variables, whose comparison in the same subject in different situations was performed using the Friedman test in the R Studio (Version 2022.12.0.0 Posit Software, PBC, USA), Version 19.1.3, with an adjusted level of significance at 0.05. Post hoc comparisons using Bonferroni correction were used to indicate the mean score for different conditions.

The Friedman test was chosen because of its non-parametric nature, applicability to related samples, tolerance for ordinal data, and robustness to outliers. All these requirements are aligned with the characteristics of single-subject designs. In our study, we collected data from the same patient under various conditions, often on an ordinal scale. This test also provides the flexibility to perform post hoc tests for pair-wise comparisons when significant differences are detected, enabling one to pinpoint where these differences exist.

### 2.6. Data Analysis

To investigate the cortical effects using our BCI, we analyzed the significant event-related desynchronization (ERD) patterns in the time-frequency representation of the EEG signals. Additionally, we performed Pearson correlation analyses between cycling velocities in the Mu, low beta, and high beta rhythms.

The significant ERD pattern analysis only involved successful pedal activation through MI trials with passive movement feedback. We extracted 7-s segments aligned to the MP’s onset movement (being 0 s the onset). Each segment included a 2-s baseline period preceding MI recognition (−3 to −1 s), a 1-s MI period (−1 to 0 s) for pedal activation, and a 4-s period after MI recognition representing passive movements (0 to 4 s). EEG data from −2.0 to −1.0 s served as a reference or baseline, whereas data from 0 to 3 s represented cortical activity during passive pedaling (PP). The segments were first band-pass filtered from 0.1 to 40 Hz and later analyzed through the t-percentile bootstrap algorithm to determine significant ERD patterns, considering significance levels of 0.01 and 0.20 (confidence intervals of 99% and 80%, respectively).

## 3. Results

### 3.1. Functional and Somatosensory Parameters

A summary of the data obtained through functional evaluation is presented in Table 1. To evaluate gait and distinguish between baseline and the last assessment, a 10-m walking test was used. We observed a reduction of 39.99% in the number of steps per minute using this test at the last assessment. On the other hand, notable changes in the physical and motor function of the lower-limbs were identified using the Fugl-Meyer Assessment (FMA) weekly in relation to the baseline. Additionally, we observed a gradual increase in the motor function, coordination, and speed of the affected limb; however, none of these differences was statistically significant.

The Semmes Weinstein Monofilaments test was used to evaluate the somatosensory parameter, whose results are described in Table 2. In general, we found the recovery of protective sensitivity and discrimination of shapes, temperature, and texture at L1 (coxofemoral joint) and S2 (popliteal fossa), in addition to recovery from incapacity to diminished protective sensation at L2 (medial thigh region) and L4 (medial malleolus).

### 3.2. Quantitative EEG

**Figure 3:**
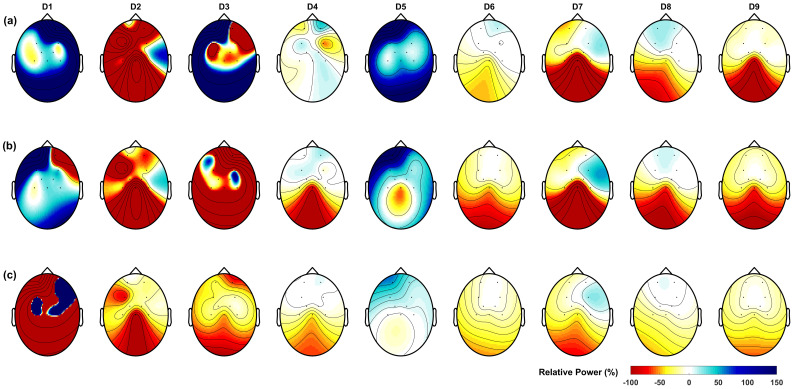
Event-related desynchronization (ERD) analysis using the time-frequency representation. The ERD patterns were analyzed in Matlab after each protocol session. The distribution of ERD changes in the time-frequency representation over Cz and topographic maps of the mean ERD power obtained while the patient was pedaling the MP for (**a**) the 8–12 Hz, Mu band; (**b**) 13–22 Hz, low beta band; and (**c**) 23–35 Hz, high beta band. A decrease in relative power (ERD) implies increased neural activity in that frequency band.

The ERD patterns were analyzed in Matlab after each protocol session (Figure 3). As a result, we identified desynchronization in the following rhythms: Mu (8–12 Hz) at the regions FP1, FP2, F3, F4, FC1, FC2, and C4; low beta (13–22 Hz) at FP1, F3, F4, FC1, FC2, C3, C4, and Cz; and high beta (23–35 Hz) at FP2, F3, F4, FC1, and C4. We also found ERS patterns in the Mu rhythm at the region C3; in low beta at FP2; and in high beta at FP1, C3, and Cz.

**Figure 4:**
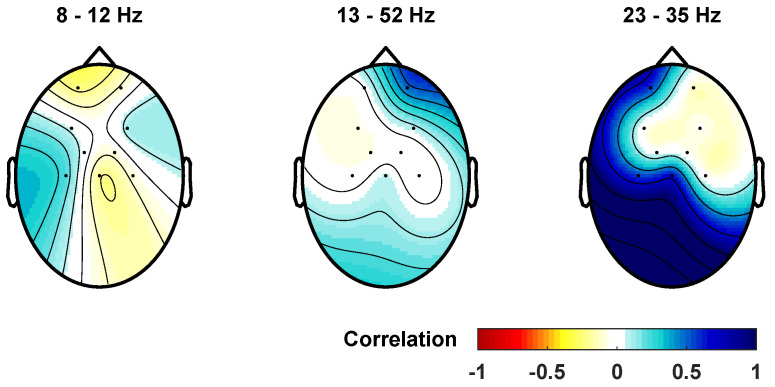
Pearson correlation between the velocities of cycling and rhythms 8–12 Hz (Mu), 13–22 Hz (low beta), and 23–35 Hz (high beta). The Pearson correlation coefficient measures how closely two variables are related on a scale from −1 (red) to 1 (blue), where 0 (white) represents no linear correlation.

We also tested the Pearson correlation between the velocities of cycling rhythms in Mu, low beta, and high beta (Figure 4). As a result, we found a positive correlation between high beta in FP1, C3, and Cz; low beta in FP1, FP2, F4, and Cz; and Mu in F3, F4, FC1, and C3. We also found a negative correlation in high beta at FP2, F3, F4, FC2, and C4; in low beta in F3, FC1, FC2, C3, and C4; and Mu in FP1, FP2, FC2, C4, and Cz.

## 4. Discussion

We investigated the sequential use of tDCS and MI-BCI, as well as the use of a VR and an MP device, for neurological recovery in people with a loss of motor function due to stroke. The aim of this research was to measure and compare the effects of tDCS in a post-stroke patient. Overall, we report two important results: first, an improvement in somatosensory parameters; second, a correlation between cycling velocities and the Mu, low beta, and high beta rhythms. However, despite the good results that we have obtained, as our findings are based on only one subject, the results of such analyses should be treated with considerable caution.

The existing literature on tDCS has primarily focused on upper-limb rehabilitation [5], whereas our protocol uniquely targets lower-limb restoration and involves both real and virtual actuators. It is widely recognized that various tDCS stimulation parameters, including the electrode characteristics, location, size, polarity, intensity, and duration, influence its effects [25]. Our results indicate that tDCS, with the cathode over the unaffected cerebellum, enhances the cortical excitability in the primary motor cortex (M1) of the affected hemisphere due to its depolarizing effects on neuronal resting membrane potentials. Additionally, researchers have found that tDCS over the cerebellum affects cortical excitability in a polarity-specific manner, with the predominant cerebellar neuron, the Purkinje cell, being excited when the cathode is placed over the cerebellum [26,27]. However, to the best of our knowledge, the cerebro-cerebellar pathway, employing simultaneous anodal placement on M1 and cathodal placement on the contralateral cerebellum, remains understudied in post-stroke subjects. It is worth mentioning that electromechanics-assisted gait training, particularly in combination with physical therapy, has been effective in promoting gait recovery in post-stroke patients, particularly those in the early post-stroke phase or initially unable to walk. On the other hand, the VR device allows for a motivating environment for task-specific practice, potentially enhancing rehabilitation engagement, especially in cases where motivation is crucial [25].

Assessing post-stroke body function and structure is crucial in quantifying motor impairments, predicting outcomes, monitoring recovery and therapy responses, and guiding treatment decisions. Valid and standardized measures are essential in assessing various aspects, including neural systems, gait velocity for leg motor deficits, and somatosensory deficits. The FMA quantitatively evaluates motor and sensory impairment in post-stroke subjects and, in our study, it showed gradual improvements in coordination and movement speed on the hemiparetic side, likely due to active locomotion using our BCI and physical training. We believe that combining this with tDCS in our protocol potentiated brain plasticity. Our results differ from a related study [28], which revealed significant motor function improvements post-intervention in both the MI-BCI and MI-BCI + tDCS groups, but without significant group differences. However, the gait velocity and cadence decreased, highlighting the challenges of post-stroke ambulation. Balance, assessed with the MiniBESTest score, demonstrated a 50% improvement, vital for activities of daily living. Balance impairments, stemming from sensory system deficits, underscore the importance of detection and intervention to prevent undesirable consequences [29,30,31].

The Semmes Weinstein Monofilaments test was employed to assess the somatosensory parameter, offering an inexpensive, non-invasive, and user-friendly means of quantifying skin pressure detection thresholds. Our results demonstrated the recovery of protective sensitivity, discrimination of various sensory attributes, and transitions from incapacity to diminished protective sensation at specific sites. Sensory impairments after stroke, spanning various modalities, including tactility, pain, temperature, pressure, vibration, proprioception, stereognosis, and graphesthesia, are closely tied to activity limitations and participation restrictions [32]. These impairments, as suggested by the American Heart Association [31], can be improved with therapeutic interventions, notably those encompassing multimodal approaches such as VR and augmented reality. In a parallel study, the effects of tDCS were examined during the first-month post-stroke onset, extending observations over the initial year. Acute stroke patients received either anodal tDCS or sham tDCS alongside conventional rehabilitation for 20 sessions over four weeks. Electrodes were strategically placed, with significant effects noted for “time” and “time by treatment” in various outcomes at the one-year follow-up, although the Semmes Weinstein test and the somatosensory section of the FMA did not exhibit significant differences [33].

Clinical evidence supports tDCS’ efficacy in enhancing muscular strength, motor coordination, and mobility, emphasizing its non-invasive potential in optimizing post-stroke neuromuscular rehabilitation and improving life quality [5]. In EEG analysis, specific neural oscillatory bands reveal insights into tDCS and BCI applications. Mu band analysis suggests that anodal tDCS over the affected hemisphere’s motor cortex increases excitability, also increasing desynchronization linked to attention and MI. On the other hand, cathodal placement over the unaffected cerebellum enhances inter-hemispheric connectivity and cerebellar modulation, increasing synchronization. In the low beta band, anodal tDCS enhances sensory processing, raising synchronization. In the high beta band, tDCS and BCI induce cortical inhibition and desynchronization in select regions, potentially regulating motor activity. Another study [34] examined the effect of tDCS and transcranial alternating current stimulation (tACS) on ERD during MI, finding significant differences in Mu and beta rhythms, highlighting the potential for tDCS to modulate ERD and motor-related brain activity.

tDCS is a neuromodulatory technique gaining prominence in the realm of neuromuscular rehabilitation post-stroke. Through the application of low-intensity electrical currents to the cerebral cortex, tDCS engenders neuronal membrane polarization, thereby creating alterations in cortical excitability within specific brain regions. Mainly targeting the motor cortex, tDCS endeavors to induce synaptic plasticity, thereby facilitating the restructuring of neural pathways disrupted by stroke [7]. This induced neuroplasticity through tDCS is conducive to the establishment of novel synaptic connections and the restoration of compromised motor functions. Clinical investigations have substantiated the efficacy of tDCS in ameliorating the physical functionality of stroke survivors, attaining noteworthy enhancements in muscular potency, motor coordination, and overall mobility [5]. These affirmative outcomes underscore the non-invasive and promising nature of tDCS as an intervention, holding substantial potential in optimizing post-stroke neuromuscular rehabilitation and, in turn, allowing improved life quality for afflicted individuals.

In the context of EEG analysis during tDCS and BCI application, distinctive neural oscillatory bands are scrutinized for insights into the neural mechanisms at play. Mu band analysis reveals that positioning the anode over the affected hemisphere’s primary motor cortex potentially increases excitability in relevant regions, augmenting desynchronization tendencies, and is possibly linked to bolstered attention and motor imagery processes. Concurrently, cathodal placement over the cerebellum of the unaffected hemisphere might exert inter-hemispheric connectivity effects and cerebellar modulation over motor function, whose concerted influence results in augmented synchronization within the Mu band. Likewise, in the low beta band, anodal tDCS placement appears to modulate cortical excitability, concomitantly raising synchronization levels, possibly associated with enhanced sensory processing. In the high beta band, the combined effect of tDCS and BCI is projected to induce cortical inhibition, instigating desynchronization within select cortical regions, potentially related to motor activity regulation. Notably, cathodal placement over the unaffected hemisphere’s cerebellum may facilitate functional integration between pertinent regions, leading to elevated synchronization. To summarize, the combination of tDCS and BCI, with specific montage arrangements, exerts a discernible influence on diverse brain oscillatory patterns, leading to distinctive synchronization and desynchronization responses, as delineated in our research.

Despite noticeable progress in functional recovery, statistical significance in terms of both ambulatory and sensory outcomes was not achieved. In addition, an important limitation of this study was the evaluation of only one subject. However, it is important to consider the context in which the study was conducted, during the height of the COVID-19 pandemic, which influenced the chosen methodology. Nevertheless, this preliminary study is still valuable as it provides a foundation for future studies involving a larger and more diverse participant group.

A potential pathway to explore for the promotion of a wider range of neurophysiological engagement conducive to enhancing functional recovery is to augment the frequency and duration of the training sessions over several weeks. This strategic intensification may have the potential to enhance the inherent neurological modulatory effects of tDCS, which could lead to more substantial advancements in facilitating rehabilitative progress related to ambulation, for example.

Importantly, it should be noted that interruptions occurred during some protocol sessions, as the participant had urinary incontinence and discomfort caused by motion sickness while using the VR glasses.

## 5. Conclusions

The use of low-cost robotic devices for stroke rehabilitation can optimize therapy and reduce the total cost of the rehabilitation process, which includes health and rehabilitation services, specific projects aimed at the labor market, education and professional training, social benefits, the distribution of assistive devices, and urban mobility. In this study, we aimed to promote scientific and technical knowledge about technologies in neuromuscular rehabilitation for those with severe motor impairment and make this technology accessible to the population.

This research aimed to explore a protocol for neurorehabilitation and measure its effects on post-stroke patients through the use of tDCS and MI-BCI, together with a VR and an MP device. Our hypothesis was that there would be an improvement in the physical function and brain activity of post-stroke patients after using our protocol.

Based on our results, we cannot conclude that adjuvant mental practice training is superior to conventional occupational therapy alone in subacute post-stroke patients. However, based on the results obtained so far, we propose conducting new studies with a larger sample size to further examine this.

## Figures and Tables

**Table 1:** Functional parameters for the assessed subject.

Parameter	First Assessment	Second Assessment	Change (%) First and Second	Third Assessment	Change (%) First and Third	Fourth Assessment	Change (%) First and Fourth
Gait Velocity (m/s)	0.16	0.11	−31.25	0.13	−18.75	0.15	−6.25
Cadence (steps/m)	29.33	25	−14.76	22.66	−22.74	17.6	−39.99
MiniBest Test	2	1	−50	3	50	3	50
FM Lower Extremity (Affected side)	6	6	0	7	16.66	9	50
FM Lower Extremity (Non-affected side)	13	12	−7.69	16	23.07	16	23.07
FM Coordination and Velocity	4	4	0	5	25	6	50
FM Balance	6	4	−33.33	3	−50	6	0

**Table 2:** Mean evolution of every somatosensory parameter measured over the time course of our study.

Dermatome Levels	First Assessment (g)	Second Assessment (g)	Third Assessment (g)	Fourth Assessment (g)
T10	2	2	2	2
T12	2	0.2	0.2	2
L1	10	2	4	2
L2	4	0.2	0.2	2
L3	2	4	4	2
L4	10	300	2	2
L5	300	10	10	300
S1	4	4	4	4
S2	4	0.2	0.2	2

We can interpret the target in grams (g) in the following way: 0.05 g as normal sensation in the hand and foot; 0.2 g as decreased sensitivity and difficulty discriminating texture (light touch); 2.0 g as decreased protective sensitivity in the hand, inability to discriminate texture, and difficulty discriminating shapes and temperature; 4.0 g as loss of protective sensation in the hand and sometimes the foot, loss of texture discrimination, and inability to discriminate shapes and temperature; 10 g as loss of protective sensation in the foot, texture discrimination loss, and inability to discriminate shapes and temperature; and 300 g as only the feeling of deep pressure remaining. The parameters were evaluated according to the dermatome levels.

## Data Availability

The data that support the findings of this study are available upon reasonable request from the authors.
